# Artificial Host‐Guest Recognition Directs Glycometabolically Engineered Macrophages to Tumors

**DOI:** 10.1002/advs.76468

**Published:** 2026-07-13

**Authors:** Zhiqing Yang, Qian Cheng, Ziyi Wang, Jianwen Wei, Qun Guan, Huazhong Yu, Qingwen Zhang, Caixia Yin, Ruibing Wang

**Affiliations:** ^1^ State Key Laboratory of Mechanism and Quality of Chinese Medicine Institute of Chinese Medical Sciences University of Macau Macau Macao SAR China; ^2^ MoE Frontiers Science Center for Precision Oncology University of Macau Macau Macao SAR China; ^3^ Chinese Medicine Germplasm Resources Innovation and Effective Uses Key Laboratory of Sichuan Province Chengdu University of Traditional Chinese Medicine Chengdu China; ^4^ School of Traditional Chinese Medicine Faculty of Medicine Yangzhou University Yangzhou China; ^5^ Department of Chemistry Simon Fraser University Burnaby British Columbia Canada; ^6^ Key Laboratory of Chemical Biology and Molecular Engineering of Ministry of Education Institute of Molecular Science Shanxi University Taiyuan China

**Keywords:** glycometabolic labeling, host‐guest recognition, macrophage engineering, tumor‐targeting

## Abstract

CD3ζ‐based chimeric antigen receptor macrophage (CAR‐M) therapy for solid tumors is limited by complex viral‐mediated genetic engineering and the challenge of maintaining a durable pro‐inflammatory phenotype within the immunosuppressive tumor microenvironment. Here, we introduce a supramolecular glycoengineering strategy that bypasses genetic modification. By utilizing a novel metabolic labeling agent, Ac_4_ManNAda, we successfully install adamantane (Ada, a guest molecule) tags onto macrophage surfaces via native biosynthetic pathways to generate glycoengineered supramolecular macrophages (GSAR‐M). This labeling not only significantly enhances recognition of β‐cyclodextrin (β‐CD, a host molecule)‐tagged tumor cells via CD‐Ada host‐guest interactions but also, unexpectedly, acts as an intrinsic activator. It induces a sustained distinct activated state in GSAR‐M, characterized by upregulated activation markers and enhanced migratory, phagocytic, and tumoricidal capacities. Furthermore, subsequent LPS stimulation of these cells (termed GSAR‐M+) cooperatively amplifies F‐actin content and pseudopodia formation, leading to superior tumor cell capture in vitro. In a murine 4T1 breast cancer model, this supramolecular glycoengineering strategy achieves profound tumor growth arrest and effectively remodels the immunosuppressive tumor microenvironment. This study establishes a streamlined, cost‐effective, and non‐viral engineering paradigm that integrates host‐guest recognition with glycometabolic engineering, providing critical insights for the development of next‐generation engineered immune cells in adoptive cell therapies.

## Graphical abstract

Glycoengineered supramolecular macrophages (GSAR‐M) are developed using glycometabolic labeling for bioorthogonal host‐guest tumor targeting. This engineering approach unexpectedly enhances macrophage migration, phagocytosis, and pseudopodia formation. Consequently, GSAR‐M demonstrate robust tumor‐targeting specificity, effectively arrest tumor growth, and remodel the immunosuppressive tumor microenvironment in vivo, presenting a powerful strategy for adoptive cell therapy.

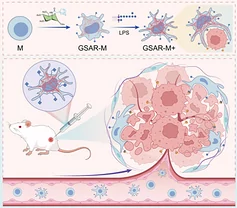

## 1 Introduction

Adoptive cell therapy refers to a therapeutic strategy where immune cells collected from patients or allogeneic donors are expanded and modified ex vivo before being reinfused back into the patient [1]. Compared to other immunotherapeutic approaches based on alleviating intrinsic immune suppression or activating endogenous immune cells within patients, the ex vivo manipulation process in adoptive cell therapy provides unique opportunities to precisely engineer immune cells for enhanced functionality. Among them, chimeric antigen receptor T‐cell (CAR‐T) therapy has demonstrated breakthrough efficacy in treating hematological malignancies [2, 3, 4]. However, its application in solid tumors remains substantially constrained by the highly immunosuppressive tumor microenvironment (TME), heterogeneous antigen expression, and limited T‐cell infiltration [5]. To address these challenges, chimeric antigen receptor macrophages (CAR‐M) have emerged as a promising alternative [6]. Macrophages not only efficiently infiltrate solid tumor tissues but also exhibit intrinsic phagocytic capability and antigen‐presenting functions, thereby potentiating immunostimulatory remodeling of the TME. Although the first‐generation CD3ζ‐based CAR‐M can phagocytose tumor cells in an antigen‐dependent manner, they fail to sustain durable M1‐like pro‐inflammatory polarization and are prone to re‐polarization toward immunosuppressive M2 phenotypes [7]. Furthermore, conventional engineering paradigms that utilize retroviral or lentiviral vectors for chimeric antigen receptor gene integration exhibit technical complexity, high costs, and potential oncogenic risks, necessitating the development of non‐viral cellular engineering approaches with simplified procedures, cost‐effectiveness, and enhanced safety profiles.

Metabolic glycan labeling of unnatural sugars refers to the cellular engineering strategy that utilizes glycometabolic engineering pathways to integrate chemically modified unnatural sugars, including mannose, galactose, fucose, etc., with proteins and ultimately expresses them in the form of glycoproteins on cell membranes [8]. Given that the chemical tags on membrane glycoproteins enable diverse bioorthogonal reactions such as click chemistry, efforts have recently been devoted to exploring the potential of this non‐viral and chemically modified unnatural sugar‐based metabolic glycan labeling for potential adoptive cell therapy, with promising results achieved in engineering immune cells including T cells [9, 10, 11, 12] and macrophages [13, 14]. Notably, limited by the molecular dimensions and steric hindrance of the chemical moieties conjugated to unnatural sugars, which can impair labeling efficiency, as well as the high toxicity of copper ions required for conventional click chemistry on cells, current in vivo applications predominantly focus on azide‐modified unnatural sugars for glycometabolic engineering, followed by reagent‐free click chemistry with dibenzocyclooctyne [15, 16, 17, 18, 19]. To unlock a new dimension in metabolic glycan labeling of immune cells, we put forward a supramolecular guest molecule as a compelling candidate for glycan labeling, which will allow host‐guest interaction‐initiated recognition.

These highly selective, strong yet dynamic supramolecular host‐guest interactions have recently served as versatile building blocks for bioorthogonal diagnostic and therapeutic applications, including multiplex bioimaging [20, 21], targeted delivery of antimicrobial and anticancer drugs [22, 23, 24, 25, 26, 27, 28], spatiotemporally controlled activation of prodrugs in vivo [29, 30, 31, 32], intelligent regulation of supramolecularly gated nanozymes [33, 34, 35, 36], and intracellular self‐assembly of bioresponsive nanomaterials [37, 38, 39, 40, 41]. Given this versatility, integrating supramolecular chemistry into glycometabolic cell labeling offers new prospects for adoptive cell therapy. In particular, the favorable steric profile of adamantane (Ada) (being much less bulky than various host molecules) and its capacity for multivalent complexation with cyclodextrins and cucurbiturils makes it an ideal candidate to overcome the key bottlenecks of current sugar tags, thereby unlocking new dimensions in metabolic glycan labeling of cells.

Here, we report glycoengineered supramolecular macrophages (termed GSAR‐M) that are glycometabolically labeled with guest molecule Ada for the first time, allowing for tumor targeting via bioorthogonal, artificial host‐guest interactions following adoptive transfer. Specifically, N‐acetylmannosamine (ManNAc)‐modified adamantane, termed Ac_4_ManNAda, was synthesized for glycometabolic labeling and tracking macrophages. Through efficient host‐guest interactions, these engineered macrophages specifically recognize β‐cyclodextrin (β‐CD)‐bearing tumor cells, which serve as an in vivo pre‐targeting [42, 43, 44, 45] model. Remarkably, this Ac_4_ManNAda‐based glycometabolic engineering not only upregulates activation markers and enhances the migratory and phagocytic capabilities of macrophages but also, upon lipopolysaccharide (LPS) stimulation, yields an unexpected promotion of pseudopodia formation, further augmenting their migratory and phagocytic capabilities. Consequently, these pseudopodia‐enhanced glycoengineered supramolecular macrophages, which integrate glycometabolic labeling with a guest molecule capable of bioorthogonal host‐guest recognition, demonstrate robust tumor‐targeting specificity, profound tumor growth arrest, and the capacity to effectively remodel the immunosuppressive tumor microenvironment post‐adoptive transfer in vivo (Figure 1), highlighting a promising application of glycometabolic labeling in adoptive cell therapy.

**FIGURE 1:**
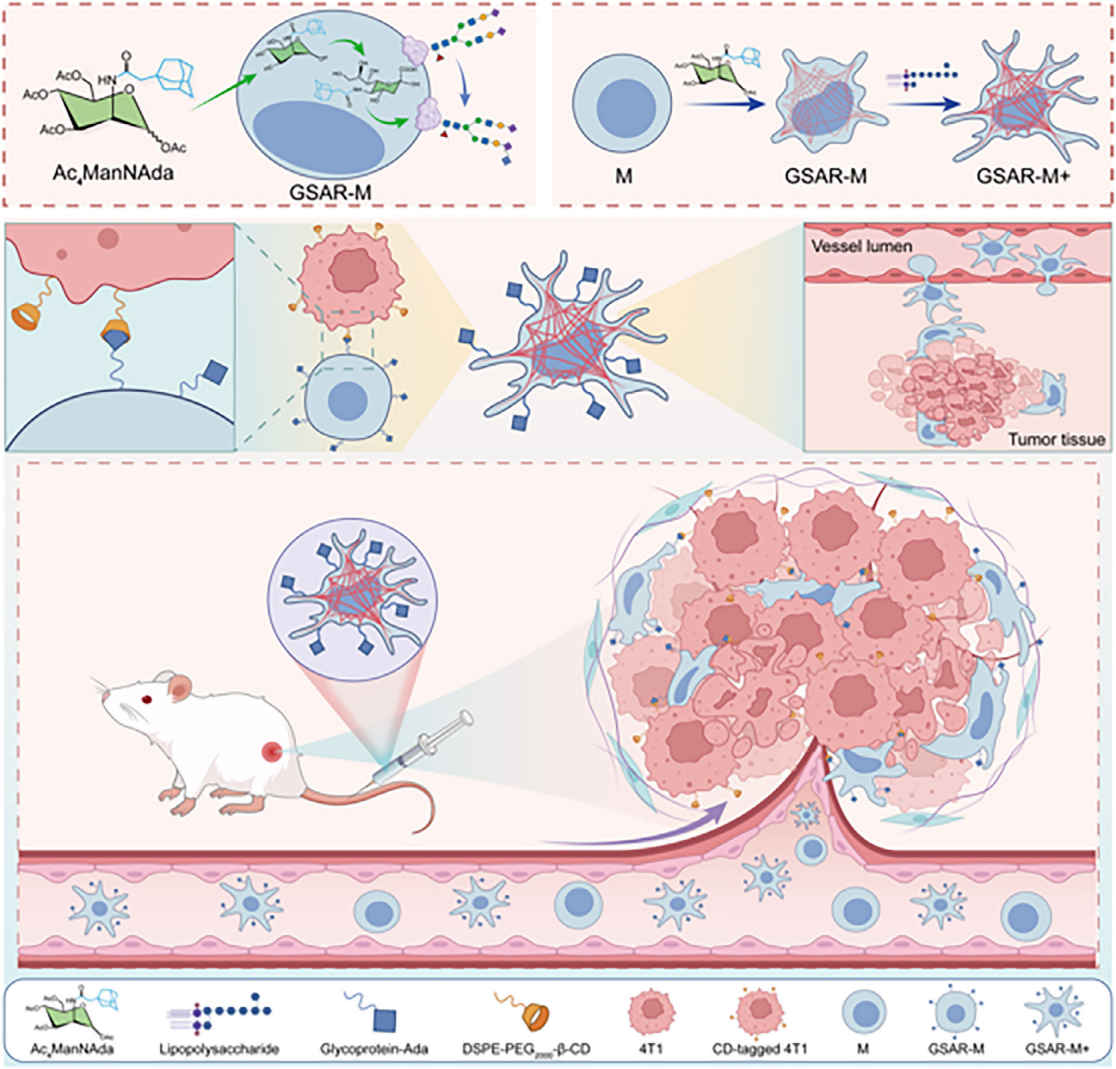
Upon glycometabolic labeling of Ac_4_ManNAda, macrophages display enhanced migratory and phagocytic abilities, while gaining tumor‐targeting capability via host‐guest recognition of β‐CD‐bearing tumor cells. This priming effect is further amplified by LPS stimulation, markedly promoting pseudopodia formation, ultimately leading to robust antitumor efficacy upon adoptive transfer.

## 2 Results and Discussion

### 2.1 Preparation of GSAR‐M

To achieve the supramolecular engineering of macrophages with the guest molecule Ada via metabolic glycoengineering, we synthesized 1,3,4,6‐tetra‐O‐acetyl‐N‐(1‐adamantylacetyl)‐D‐mannosamine (Ac_4_ManNAda) for the first time. The synthesis was adapted from the established route for the classic metabolic labeling agent tetraacetyl‐N‐azidoacetylmannosamine (Ac_4_ManNAz) [46], substituting the azido‐modification with an adamantane moiety. The product was obtained as a mixture of anomers. HPLC analysis revealed two peaks at retention times of 8.26 and 8.38 min, with peak areas of 22.9% and 76.5%, respectively, corresponding to the α‐ and β‐anomers (Figure S1 and Table S1). Consistent with the HPLC result, the ^1^H NMR spectrum also exhibited two sets of signals corresponding to the mixture of anomers (Figure S2). The LC‐MS spectrum exhibited a base peak at *m/z* 546.3 [M + Na]^+^, along with characteristic in‐source fragmentation ions at *m/z* 464.4, 404.3, 344.3, and 284.4. These correspond to the sequential loss of four acetic acid molecules (60 Da), specifically [M + H − HOAc]^+^, [M + H – 2HOAc]^+^, [M + H – 3HOAc]^+^, and [M + H – 4HOAc]^+^, which confirms the presence of the tetra‐acetylated structure (Figure S3 and Table S2). Collectively, HPLC, ^1^H NMR, and LC‐MS analyses demonstrated the successful synthesis of Ac_4_ManNAda.

Given that the sialic acid biosynthetic pathway can accommodate specific bulky modifications such as norbornene [47], dibenzocyclooctyne [48], and 2,4‐dinitrophenyl groups [49], we hypothesized that the adamantane moiety could be similarly processed. As schematically outlined in Figure 2A, our strategy is predicated on the rational design that Ac_4_ManNAda can be taken up by macrophages and metabolically converted to SiaNAda, leading to its ultimate presentation on the cell surface. To validate this design and confirm the surface display, DSPE‐PEG_2000_‐β‐CD‐modified liposomes loaded with fluorescein isothiocyanate (CD‐FITC lipo) were used to probe the cell‐surface adamantane based on the host‐guest interaction between cyclodextrin and adamantane. The rapid kinetics of host‐guest complex formation allowed for an ultra‐short 5‐min incubation with the cells, thereby minimizing nonspecific cellular uptake. Using this probing strategy, the metabolic incubation time was first optimized. The conventional incubation time for metabolic engineering with unnatural sugars (e.g., Ac_4_ManNAz) reported in the literature typically ranges from 1 to 3 days. Based on this time window, we focused our validation on 24 and 48 h. Flow cytometry analysis (Figures S4 and S5) revealed that the fluorescence signals on the cell surface exhibited negligible enhancement following 24 h of incubation with Ac_4_ManNAda. In contrast, extending the incubation to 48 h resulted in a significant increase in green fluorescence signals across all tested concentrations. This demonstrates that a 48‐h incubation is sufficient for the efficient metabolic expression of adamantane moieties on the macrophage membrane. Therefore, 48 h was selected for subsequent preparation of GSAR‐M. In contrast to the negligible fluorescence in PBS‐treated control macrophages (M), GSAR‐M showed distinct surface‐localized green fluorescence (Figure 2B), confirming the successful glycometabolic labeling of adamantane on the macrophage membrane. Furthermore, the green fluorescence intensity on the macrophage membrane increased in a concentration‐dependent manner. Flow cytometry analysis (Figure 2C,D) demonstrated that the green fluorescence signals on GSAR‐M treated with 200, 400, and 600 µm Ac_4_ManNAda were enhanced by 1.4‐, 2.3‐, and 2.7‐fold, respectively, compared to M.

**FIGURE 2:**
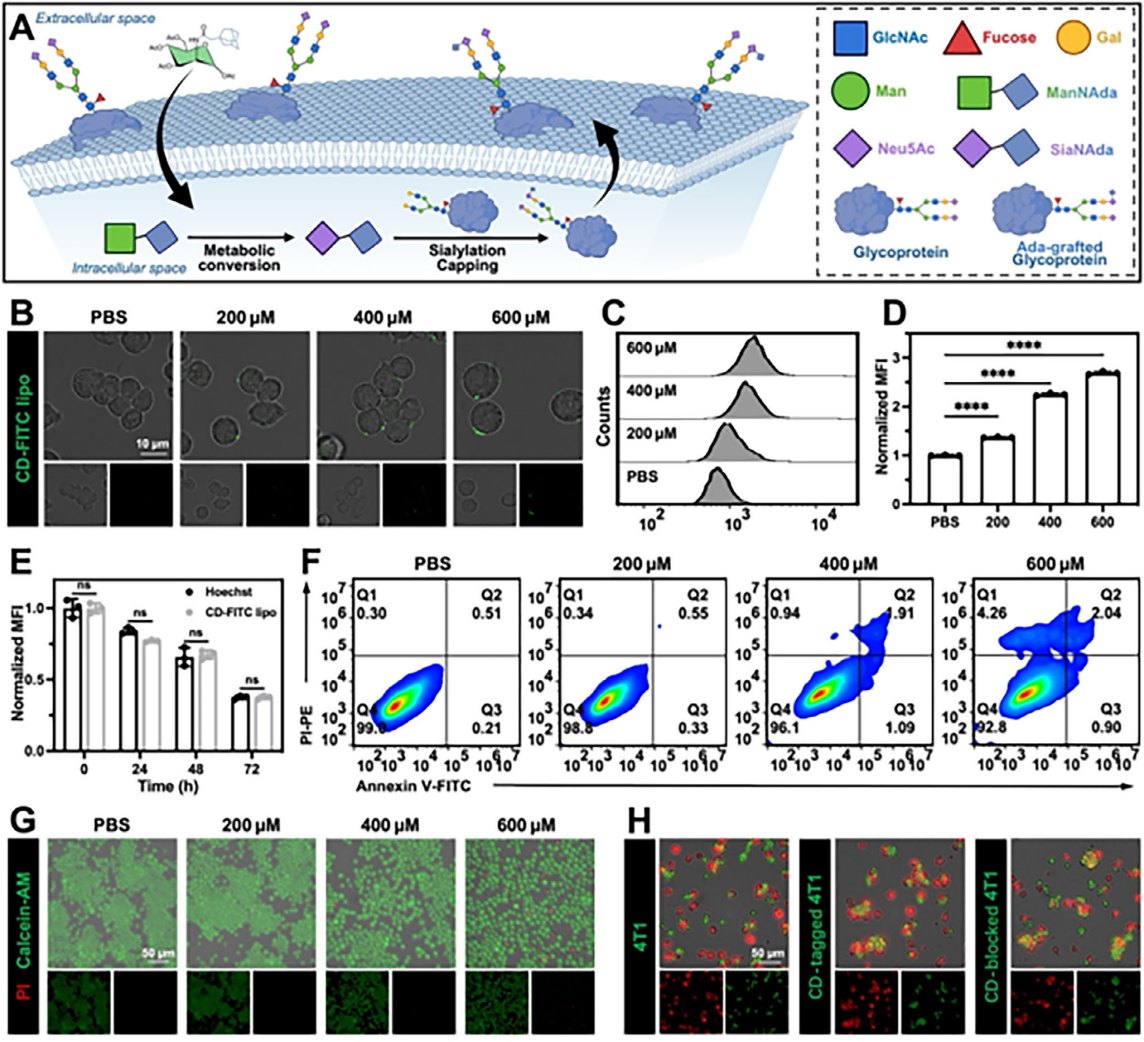
Glycometabolic engineering of macrophages and in vitro validation of host‐guest targeting. (A) Schematic illustration of glycometabolic labeling of macrophages with Ac_4_ManNAda (created using BioRender.com). (B) Confocal images, (C) flow cytometry analysis of macrophages following a 2‐day incubation with Ac_4_ManNAda and subsequent 5‐min staining with CD‐FITC lipo, (D) and the corresponding normalized mean fluorescence intensities (*n* = 3). (E) Stability of the glycometabolically labeled adamantane moiety on macrophages (*n* = 3). (F) Apoptotic rates, (G) and live/dead imaging of macrophages incubated with Ac_4_ManNAda, with the concentrations varying from 0 to 600 µm. (H) Fluorescence images of GSAR‐M (red) co‐cultured for 4 h with unmodified, CD‐tagged, or CD‐blocked 4T1 cells (green). Data are expressed as mean ± SD (D, E). Statistical significance was calculated using ordinary one‐way ANOVA followed by Dunnett's multiple comparison tests (D) and two‐way repeated measures ANOVA followed by Šídák's multiple comparison tests (E). ^*^ *p* ≤ 0.05, ^**^ *p* ≤ 0.01, ^***^ *p* ≤ 0.001, ^****^ *p* ≤ 0.0001.

Moreover, the stability of the metabolically labeled adamantane moieties on macrophages was evaluated. Specifically, Hoechst‐prestained GSAR‐M were subjected to a brief 5‐min CD‐FITC lipo incubation at 0, 24, 48, and 72 h post‐engineering. Quantitative results of flow cytometric analysis revealed a coordinated decay trend between the attenuation of nuclear Hoechst signal and the diminution of membrane‐localized FITC fluorescence (Figure 2E). This demonstrates the durable retention of adamantane moieties on the cell surface, with fluorescence attenuation primarily dominated by natural cell proliferation rather than rapid degradation or shedding.

Next, we evaluated the biosafety of Ac_4_ManNAda. The MTT assay (Figure S6) revealed a dose‐dependent decrease in the overall relative absorbance, with values of 83%, 68%, and 37% at 200, 400, and 600 µm, respectively. However, this reduction in the MTT readout did not equate to true cytotoxicity. Both cell apoptosis assays (Figure 2F) and live/dead cell imaging (Figure 2G) confirmed the absence of obvious apoptosis, necrosis, or significant cell death, even at the highest concentration of 600 µm. To determine whether the aforementioned reduction in the bulk MTT signal originated from metabolic impairment or proliferation inhibition, we conducted real‐time monitoring using an Incucyte S3 Live‐Cell Analysis System. Real‐time monitoring showed that the engineered macrophages in the field of view continued to proliferate, although their proliferation rate decelerated with increasing incubation concentrations (Figure S7). This suggests that the decreased MTT readout is more likely attributed to a suppressed proliferation rate following Ac_4_ManNAda incubation. Consequently, we designed an additional MTT assay normalized against a precise cell number gradient to evaluate single‐cell mitochondrial activity. To rigorously verify the consistency of cell numbers between groups prior to the assay, whole‐well images were captured following the 4‐h attachment period, confirming identical cell densities (Figure S8). The subsequent MTT results indicated that at these identical cell counts, the readout of the GSAR‐M group was consistently slightly higher than that of the unengineered M group (Figure S9). Collectively, the decline in the bulk MTT readout reflects a temporary deceleration in cell proliferation to accommodate unnatural sugar metabolism, rather than actual cellular damage. Individual engineered cells, in fact, retain robust mitochondrial metabolic activity. Therefore, to strike an optimal balance between achieving high‐density cell‐surface labeling and preserving cellular proliferation homeostasis, the intermediate concentration of 400 µm Ac_4_ManNAda was strategically selected for subsequent preparation of GSAR‐M.

Furthermore, we assessed the host‐guest interaction‐mediated tumor cell‐targeting capability of GSAR‐M using a co‐culture system (Figure 2H). In the control group with unmodified 4T1 cells, GSAR‐M and tumor cells remained dispersed with minimal association. In contrast, when co‐cultured with CD‐tagged 4T1 cells, whose membranes were pre‐anchored with β‐CD via the insertion of DSPE‐PEG_2000_‐β‐CD, GSAR‐M exhibited extensive co‐localization, with all CD‐tagged 4T1 cells being specifically targeted and captured within the field of view. This robust targeting efficiency is primarily attributed to the collective avidity arising from multivalent host‐guest interactions at the cell‐cell interface. Although the monomeric affinity between Ada and β‐CD is moderate, and the metabolic expression of the bulky adamantane moiety is typically less efficient than that of smaller chemical reporters such as azide [50, 51] due to steric hindrance, the integration of multiple host‐guest pairs across the membrane interface facilitates the formation of a multivalent binding network. This collective interaction effectively compensates for the reversible nature of individual host‐guest pairs, ensuring stable and specific cell‐cell recognition. To verify the necessity of host‐guest recognition, cyclodextrin cavities on 4T1 cells were pre‐occupied with an excess of free amantadine. In co‐cultures with these CD‐blocked cells, the specific interaction was substantially abolished, though not completely eliminated. The observed reduction in targeting efficiency aligns with the dynamic and reversible nature of host‐guest interactions, demonstrating that the targeting capability is mediated by cyclodextrin‐adamantane host‐guest recognition. To quantitatively validate the enhanced targeting efficiency observed in the confocal imaging, we performed flow cytometry analysis [7, 52] on the co‐culture system. Consistent with the co‐culture observations, the flow cytometry scatter plots and the corresponding statistical analysis revealed a significantly higher percentage of double‐positive events in the group co‐cultured with CD‐tagged 4T1 cells compared to both the unmodified and CD‐blocked control groups (Figures S10 and S11). These robust quantitative data firmly substantiate that multivalent host‐guest interactions drive the specific targeting of GSAR‐M to tumor cells.

### 2.2 Functional Activation of GSAR‐M

During the collection of GSAR‐M following glycometabolic engineering, we serendipitously observed their unusually strong adhesion to culture dishes. This unexpected physical behavior prompted us to investigate whether the Ac_4_ManNAda‐based engineering process induced broader phenotypic alterations. Flow cytometric analysis revealed that CD86 expression was significantly upregulated while CD206 was downregulated, indicating that GSAR‐M transitioned into an activated state. This state was maintained for at least 48 h, a duration highly advantageous for sustaining cellular readiness upon adoptive transfer into immunosuppressive microenvironments (Figure 3A,B). Notably, while sharing these typical surface markers with classical M1 polarization, our subsequent functional evaluations revealed this to be a distinct activation profile that is functionally skewed toward enhanced migration and phagocytosis. When compared with LPS‐stimulated macrophages (termed M+, treated with 100 ng/mL [53] LPS for 24 h), which served as a classical biochemical activation control, GSAR‐M exhibited comparable upregulation of activation markers CD80 and CD86, alongside a robust enhancement in the expression of adhesion‐ and activation‐associated integrins [54, 55] CD11b and CD11c (Figure 3C,D). Furthermore, intracellular F‐actin, a cytoskeletal component critical for cellular motility [56], was notably upregulated upon Ac_4_ManNAda treatment, supporting an enhanced migratory potential (Figure 3F).

**FIGURE 3:**
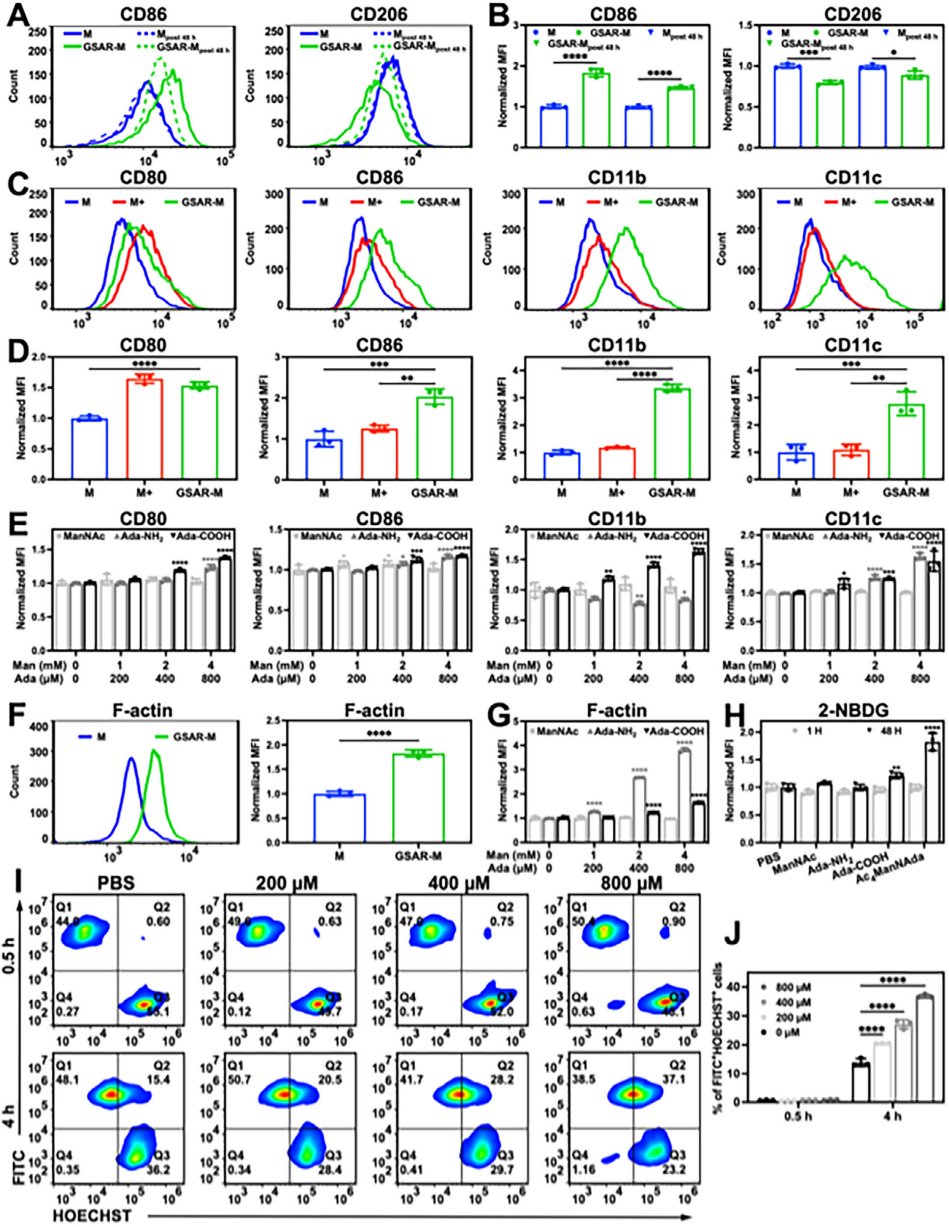
(A) Expression levels of CD86 and CD206 on macrophages after incubation with PBS or Ac_4_ManNAda for 2 days, followed by an additional 2‐day incubation with PBS, (B) and the corresponding normalized mean fluorescence intensities of them using flow cytometric analysis (*n* = 3). (C) Expression levels of CD80, CD86, CD11b, and CD11c on macrophages after treatment with different formulations, (D) and normalized mean fluorescence intensities of them using flow cytometric analysis (*n* = 3). (E) Expression levels of CD80, CD86, CD11b, and CD11c on macrophages after treatment with ManNAc, Ada‐NH_2_, or Ada‐COOH using flow cytometric analysis (*n* = 3). (F) Flow cytometric analysis of F‐actin content in macrophages after incubation with Ac_4_ManNAda for 2 days, and the corresponding normalized mean fluorescence intensities (*n* = 3). (G) F‐actin content in macrophages after treatment with ManNAc, Ada‐NH_2_, or Ada‐COOH using flow cytometric analysis (*n* = 3). (H) Uptake levels of 2‐NBDG in macrophages after treatment with PBS, ManNAc, Ada‐NH_2_, Ada‐COOH, or Ac_4_ManNAda using flow cytometric analysis (*n* = 3). (I) Flow cytometric analysis of a co‐culture experiment between Ada‐NH_2_ pre‐incubated macrophages and 4T1 cells for 4 h, (J) and the corresponding percentage of FITC^+^Hoechst^+^ cells (*n* = 3). Data are expressed as mean ± SD (B, D, E, F, G, H, J). Statistical significance was calculated using unpaired two‐tailed Student's t‐test (F), ordinary one‐way ANOVA followed by Tukey's multiple comparison tests (B, D), two‐way ANOVA followed by Dunnett's multiple comparison tests (E, G, H), and two‐way ANOVA followed by Tukey's multiple comparison tests (J). ^*^ *p* ≤ 0.05, ^**^ *p* ≤ 0.01, ^***^ *p* ≤ 0.001, ^****^ *p* ≤ 0.0001.

To ascertain the nature of this activation, a glucose uptake assay [57] was conducted. A brief 1‐h incubation with Ac_4_ManNAda did not acutely alter glucose consumption, whereas a 48‐h metabolic incorporation period significantly promoted glucose uptake (Figure 3H). This indicates that the functional enhancement is not a transient response to chemical stimuli, but rather a stable phenotypic shift accompanying the metabolic engineering process.

Given that Ac_4_ManNAda comprises a ManNAc scaffold and an Ada moiety, we sought to decouple their individual contributions. To account for the intrinsically lower cellular uptake of non‐acetylated sugars, macrophages were incubated with an elevated concentration gradient of ManNAc (up to 4 mm), representing a five‐fold increase relative to the standard testing range. MTT assays confirmed that macrophages exposed to the highest working concentrations maintained a relative absorbance of at least 70% (Figure S12), complying with ISO 10993–5 non‐cytotoxicity standards. And even at these substantially elevated concentrations, the sugar scaffold failed to elicit upregulation in activation markers. Conversely, to evaluate the functional role of the adamantane moiety, the water‐soluble derivatives Ada‐NH_2_ and Ada‐COOH were employed across the standard 0–800 µm gradient. This selection was necessitated by the inherently poor water solubility of the unmodified adamantane cage. Notably, both derivatives successfully recapitulated the overall activation trends, albeit to varying degrees, including the upregulation of CD11b and CD11c, elevation of F‐actin, and promotion of glucose uptake (Figure 3E,G,H). Because these water‐soluble derivatives trigger similar responses without metabolically integrating into the cell membrane, this phenotypic activation cannot be solely attributed to the sugar scaffold. Rather, it indicates that the unique adamantane cage structure is essential for inducing this phenotypic shift. Notably, a brief 1‐h incubation failed to acutely alter cellular glucose uptake (Figure 3H), whereas the 48‐h exposure resulted in profound functional enhancements. This temporal dependency suggests that the Ada‐driven activation is not an immediate acute physical reaction, but rather involves a sustained cellular response. While the precise molecular targets and intracellular signaling mechanisms responsible for this phenomenon remain unclear and warrant future investigation, this serendipitous finding provides a highly beneficial activated macrophage phenotype for subsequent adoptive transfer. Finally, to verify that this activated state functionally translates to improved cellular interactions, Ada‐NH_2_ was selected as a representative structural model to pre‐incubate macrophages prior to co‐culture with 4T1 cells (Figure 3I,J). The positive correlation between the proportion of FITC^+^Hoechst^+^ cells and the concentration of Ada‐NH_2_ used for pre‐incubation confirmed that this material‐driven cytoskeletal enhancement inherently potentiates the tumor‐capture capabilities of macrophages. To identify this unique activation profile, we further quantified nitric oxide (NO) production, a hallmark of classical M1 pro‐inflammatory polarization, using the Griess assay. Prior to the supernatant analysis, real‐time imaging confirmed consistent cell densities across all pre‐incubation groups while capturing the corresponding morphological shifts (Figure S13). Notably, a two‐day pre‐incubation with PBS, ManNAc, Ada‐NH_2_, or Ac_4_ManNAda did not spontaneously induce NO production. Furthermore, following a subsequent 24‐h LPS stimulation, both the direct visual comparison of the Griess reaction in Eppendorf tubes (Figure S14) and the quantitative analysis (Figure S15) demonstrated that macrophages pre‐incubated with Ada‐NH_2_ or Ac_4_ManNAda produced equivalent NO levels, which were slightly lower than the corresponding levels in the PBS or ManNAc groups. Therefore, rather than triggering a strict M1 polarization, the absence of a classical NO burst, combined with the observed upregulation of adhesion integrins, intracellular F‐actin enrichment, and augmented tumor‐capture capacity, reveals that this supramolecular engineering induces a distinct activation profile functionally skewed toward enhanced migration and phagocytosis. Collectively, these findings demonstrate that the Ac_4_ManNAda engineering platform offers a dual advantage by installing necessary supramolecular anchors for targeting while concurrently inducing a distinct activated state, establishing an ideal biological foundation for subsequent tumor clearance.

### 2.3 Potentiated Migration and Phagocytic Capacity of GSAR‐M+ via Sequential Activation

To further potentiate the antitumor capability of macrophages, GSAR‐M were subsequently stimulated with LPS, thus generating a more potent subset of macrophages termed GSAR‐M+ (Figure 4A). To assess the potential impact of LPS stimulation on the stability of glycometabolically labeled Ada on the cell surface, macrophages were sequentially incubated with Ac_4_ManNAda for 48 h and LPS for 24 h, then stained with CD‐FITC lipo to evaluate Ada presentation on the cell membrane. Confocal imaging revealed that GSAR‐M+ maintained distinct green fluorescence on the cell membrane (Figure 4B), and flow cytometric analysis further confirmed no significant difference in fluorescence intensity for GSAR‐M with or without LPS treatment (Figure 4C,D), demonstrating that LPS stimulation does not compromise the stability of the glycometabolically labeled Ada moiety on macrophages.

**FIGURE 4:**
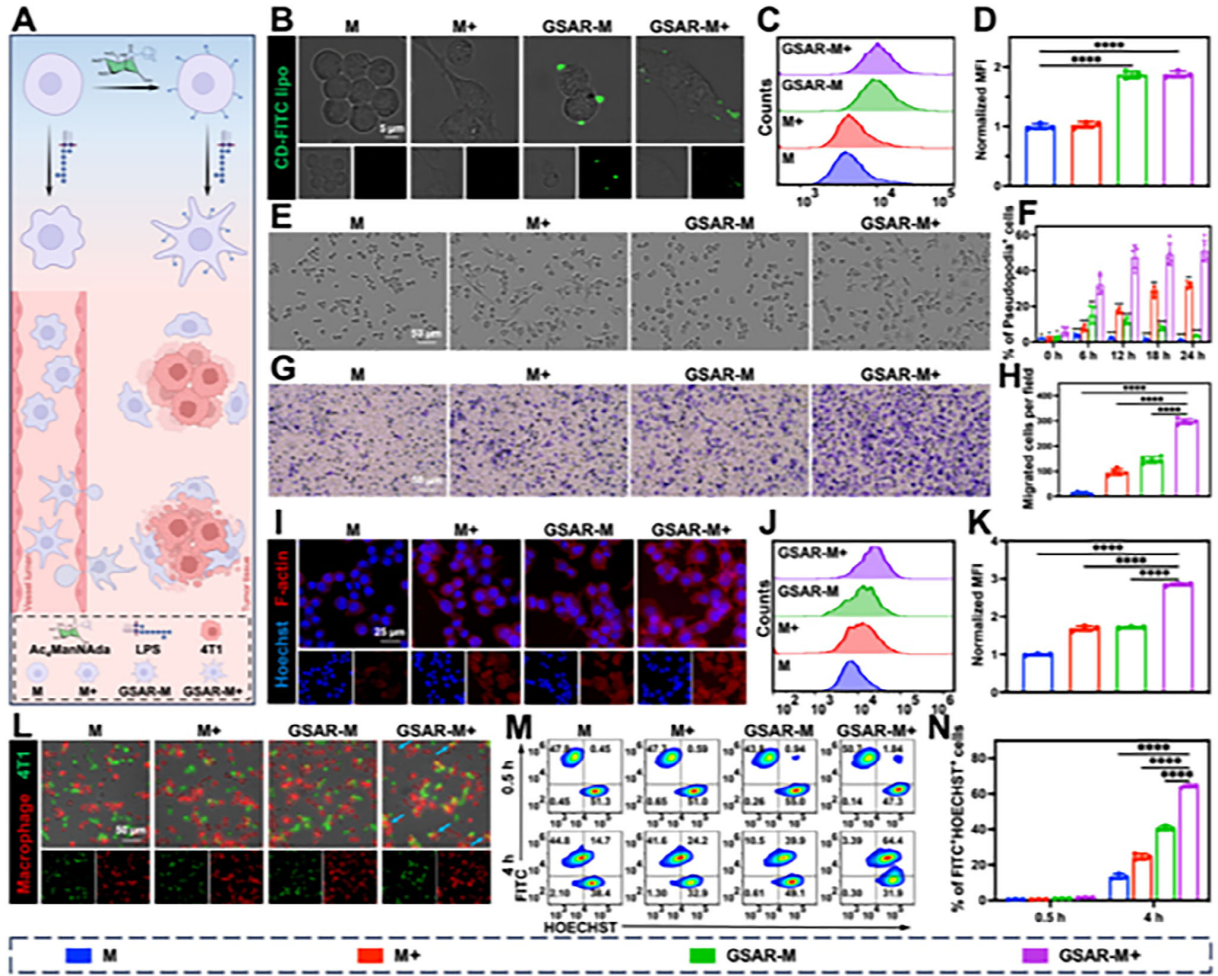
(A) Schematic illustration of the enhanced migratory and phagocytic abilities of macrophages after sequential incubation with Ac_4_ManNAda and LPS (created using BioRender.com). (B) Confocal images, (C) flow cytometric analysis of glycometabolically labeled adamantane on macrophages after incubation with PBS or Ac_4_ManNAda for 2 days, followed by incubation with PBS or LPS for 24 h, (D) and the corresponding normalized mean fluorescence intensities (*n* = 3). (E) Real‐time monitoring, (F) and quantitative analysis of pseudopodia^+^ cells after treatment with different formulations (*n* = 5). (G) Representative microscopic images of macrophages migrated through the transwell chamber, (H) and quantitative analysis of the migrated cells (*n* = 5). (I) Confocal images, (J) flow cytometric analysis of F‐actin content in macrophages after treatment with different formulations, (K) and the corresponding normalized mean fluorescence intensities (*n* = 3). (L) Fluorescence images, (M) flow cytometric analysis of macrophages co‐cultured with 4T1 cells for 4 h, (N) and the corresponding percentage of FITC^+^Hoechst^+^ cells (*n* = 3). Data are expressed as mean ± SD (D, F, H, K, N). Statistical significance was calculated using ordinary one‐way ANOVA followed by Tukey's multiple comparison tests (H), ordinary one‐way ANOVA followed by Dunnett's multiple comparison tests (D, K), and two‐way ANOVA followed by Dunnett's multiple comparison tests (F, N). ^*^ *p* ≤ 0.05, ^**^ *p* ≤ 0.01, ^***^ *p* ≤ 0.001, ^****^ *p* ≤ 0.0001.

Notably, confocal imaging revealed a striking morphological transformation in GSAR‐M+, characterized by a substantially expanded cell spreading area and the formation of extensive pseudopodia (Figure 4B). Real‐time monitoring confirmed these observations (Figure 4E), showing that GSAR‐M+ exhibited earlier pseudopod extension and a sustained higher percentage of pseudopodia^+^ cells compared to M+ under LPS stimulation throughout 24 h (Figure 4F and Figure S16).

Next, to determine whether the protruding pseudopodia enhanced macrophage migration, we conducted a transwell assay with the number of macrophages migrating outside the chamber serving as the quantification basis. As shown in Figure 4G,H, the number of macrophages migrating to the outer chamber was significantly increased in the group of GSAR‐M+, demonstrating that the protruding pseudopodia were capable of enhancing the migration ability of macrophages. Intriguingly, the GSAR‐M group, which lacked pseudopodia^+^ subpopulations, demonstrated a migration capacity comparable to the M+ group containing pseudopodia^+^ subpopulations, despite their morphological divergence.

Mechanistic investigation demonstrated that the sequential treatment of macrophages with Ac_4_ManNAda and LPS significantly enhanced F‐actin content. Fluorescence imaging showed brighter F‐actin staining, and flow cytometry confirmed a significantly increased intracellular content of F‐actin in GSAR‐M+ (Figure 4I–K). In addition, the increased intracellular F‐actin content induced by Ac_4_ManNAda alone was comparable to that induced by LPS stimulation, aligning with the trend demonstrated by the transwell assay. Based on the transient pseudopodia induction by incubation with Ac_4_ManNAda alone (Figure 4F) and the comparable final content of F‐actin between the GSAR‐M group and the M+ group (Figure 4K), we postulated that the glycometabolic labeling process of Ac_4_ManNAda enhances macrophage migration ability by promoting F‐actin formation, while subsequent LPS stimulation further facilitates macrophage motility through pseudopodia formation.

We next investigated whether this enhanced migration and motility could promote the antitumor efficacy of macrophages. Specifically, cyanine5.5 N‐hydroxysuccinimide ester (Cy5.5‐NHS)‐stained macrophages and CFDA‐SE‐stained 4T1 cells were resuspended and co‐cultured at a 1:1 ratio for 4 h, followed by imaging using a Leica DMi8 microscopy. Fluorescence imaging demonstrated greater co‐localization between GSAR‐M and 4T1 cells after 4 h of co‐culture, compared to the control group in which minimal co‐localization between M and 4T1 cells was observed (Figure 4L). Notably, GSAR‐M+ completely engulfing 4T1 cells were frequently observed and marked by blue arrows, demonstrating that the sequential incubation of Ac_4_ManNAda and LPS endowed macrophages with significantly reinforced phagocytic capacity. To maintain independence across experiments, macrophages were stained with Hoechst for flow cytometry analysis. The quantitative results also showed the highest percentage of FITC^+^Hoechst^+^ cells in the GSAR‐M+ group compared to other groups (Figure 4M,N). Collectively, these results demonstrate that the sequential integration of glycometabolic engineering and LPS stimulation endows macrophages with superior migratory and phagocytic capabilities, which are crucial functional prerequisites for effective tumor infiltration and direct cancer cell clearance.

### 2.4 In Vivo Biodistribution and Anti‐Tumor Efficacy

To evaluate the tumor‐targeting specificity of adoptively transferred macrophages enabled by the combination of glycometabolic labeling and host‐guest recognition, all macrophage formulations were stained with Cy5.5‐NHS prior to intravenous injection for in vivo tracking. IVIS imaging revealed that mice receiving an intratumoral pre‐injection of DSPE‐PEG_2000_‐β‐CD (to label tumor cells with β‐CD) followed 12 h later by an intravenous administration of GSAR‐M+ exhibited the most intense and sustained tumor‐localized fluorescence signals (Figure 5A). The fluorescence intensity at the tumor sites in this targeted group was significantly higher compared to mice receiving GSAR‐M+ alone at 48 h post‐injection (Figure 5B), demonstrating that the glycometabolic presentation of Ada enables precise tumor targeting through bioorthogonal host‐guest recognition. Furthermore, both GSAR‐M and M+ groups displayed longer retention at tumor sites compared to the M group (Figure 5A). Notably, mice receiving intravenous injection of GSAR‐M+ showed significantly higher fluorescence signals at tumor sites than mice intravenously injected with M at the 48‐h time point (Figure 5B). Consistent with our in vitro mechanistic findings, this indicates that the robust F‐actin enrichment induced by Ac_4_ManNAda and the sustained pseudopodia formation triggered by LPS collectively enhance macrophage tumor infiltration. Subsequent ex vivo imaging of harvested organs confirmed the brightest tumor‐localized fluorescence in mice receiving both the intratumoral pre‐injection of DSPE‐PEG_2000_‐β‐CD and intravenous injection of GSAR‐M+ (Figure 5C,D), validating that the integration of glycometabolic labeling and host‐guest recognition effectively achieves tumor‐targeted delivery of adoptively transferred macrophages.

**FIGURE 5:**
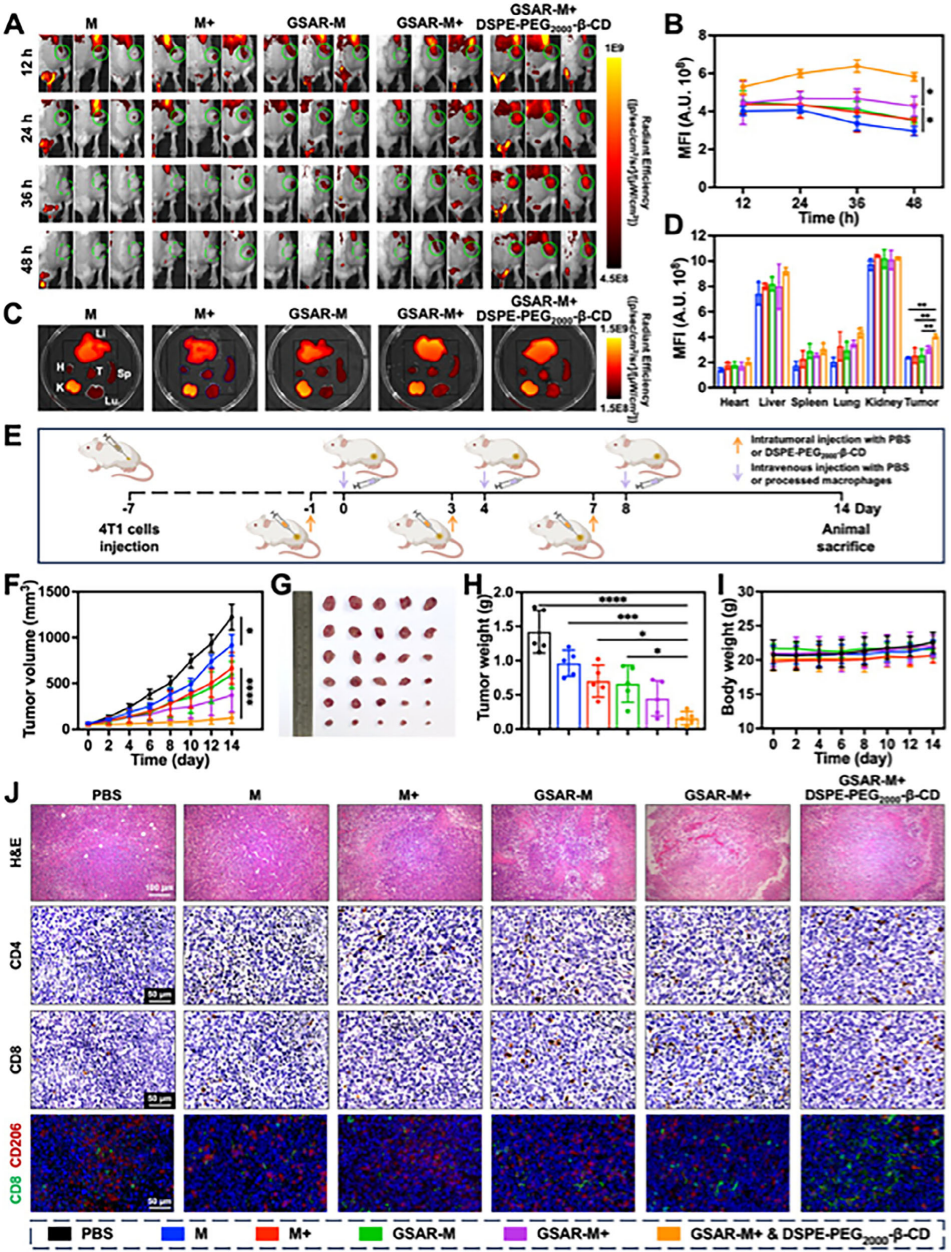
(A) IVIS images of tumor‐bearing mice after intravenous injection of Cy5.5‐tracked macrophages. (B) Mean Cy5.5 fluorescence intensities of the tumors at different time points (*n* = 3). (C) Representative IVIS images of the heart, liver, spleen, lungs, kidneys, and tumor harvested from the tumor‐bearing mice at 48 h post‐injection. (D) Mean fluorescence intensities of the organs and tumors at 48 h post‐injection (*n* = 3). (E) Schematic diagram of the experimental design (created using BioRender.com). 4T1 tumor‐bearing mice were randomly divided into 6 groups (*n* = 5) and received intravenous injections of PBS, M, M+, GSAR‐M, or GSAR‐M+ every 4 days for 3 total doses, with the final group receiving intratumoral pre‐injection of DSPE‐PEG_2000_‐β‐CD prior to GSAR‐M+ administration. (F) Tumor growth curves of mice (*n* = 5). (G) Photographs of the tumors resected at the end of the treatment. The rows from top to bottom represent the six experimental groups, namely PBS, M, M+, GSAR‐M, GSAR‐M+, and the group receiving DSPE‐PEG_2000_‐β‐CD pre‐injection before GSAR‐M+ administration. The biological replicates for each group are displayed from left to right. (H) Tumor weight (*n* = 5). (I) Body weight changes during the treatment (*n* = 5). (J) Representative images of tumor sections following histopathological and immunohistochemical analyses at the end of the treatment. Data are expressed as mean ± SD (B, D, F, H). Statistical significance was calculated using ordinary one‐way ANOVA followed by Tukey's multiple comparison tests. Notably, the statistical analyses for B, D, and F were specifically performed on the data subsets corresponding to 48 h post‐injection (B), tumor tissues (D), and day 14 (F), respectively. ^*^ *p* ≤ 0.05, ^**^ *p* ≤ 0.01, ^***^ *p* ≤ 0.001, ^****^ *p* ≤ 0.0001.

We further investigated the antitumor efficacy of these adoptively transferred macrophages. When 4T1 tumor volumes reached approximately 50 mm^3^, mice received intravenous injections of different macrophage formulations every four days for a total of three doses (with treatment protocol shown in Figure 5E). Body weight and tumor size were monitored every two days after the first dose. Tumor volume curves revealed significant tumor growth inhibition in all groups of mice receiving adoptively transferred macrophages, with moderate inhibition in the M group and greater inhibition in the M+, GSAR‐M, and GSAR‐M+ groups (Figure 5F), suggesting that the enhanced intrinsic migratory and phagocytic capacities of these macrophages led to effective tumor killing. More importantly, tumor growth was most significantly arrested in the targeted group receiving the intratumoral pre‐injection of DSPE‐PEG_2000_‐β‐CD followed by the intravenous injection of GSAR‐M+ (Figure 5G,H). This profound efficacy is attributed to the integration of the enhanced intrinsic migratory and phagocytic capacities of the engineered macrophages along with the targeting ability conferred by bioorthogonal host‐guest recognition. Moreover, no significant body weight loss was observed in any group during the treatment period (Figure 5I), indicating high systemic tolerance. Histological analysis of tumor tissues using hematoxylin and eosin (H&E) staining exhibited extensive cellular atrophy and nuclear damage across all macrophage‐administered groups, with the most severe necrosis observed in the targeted group receiving the intratumoral pre‐injection of DSPE‐PEG_2000_‐β‐CD followed by the intravenous injection of GSAR‐M+ (Figure 5J). Immunohistochemical (IHC) staining for CD4 and CD8 revealed enhanced T cell infiltration in all groups of mice receiving adoptively transferred macrophages, particularly in the groups of mice receiving intravenous injection of GSAR‐M+. Correspondingly, immunofluorescence staining further showed increased infiltration of M1‐like macrophages (CD86^+^) and decreased infiltration of M2‐like macrophages (CD206^+^) within tumor tissues across all macrophage‐treated groups, where this favorable polarization appeared most pronounced in the targeted group receiving the intratumoral pre‐injection of DSPE‐PEG_2000_‐β‐CD followed by the intravenous injection of GSAR‐M+. Collectively, these findings support that combining glycometabolic engineering with host‐guest recognition not only amplifies the antitumor efficacy of adoptively transferred macrophages but also facilitates a favorable shift in the immunosuppressive tumor microenvironment.

### 2.5 Biosafety Evaluation

To assess the biosafety of the adoptively transferred macrophages, histological analysis of major organs was conducted at the end of treatment. H&E staining showed no pathological abnormalities, lesions, or degeneration across all treatment groups (Figure 6A). Furthermore, complementary hematological analysis demonstrated that the levels of red blood cell (RBC), hemoglobin (HGB), hematocrit (HCT), mean corpuscular volume (MCV), mean corpuscular hemoglobin (MCH), mean corpuscular hemoglobin concentration (MCHC), platelet count (PLT), and mean platelet volume (MPV) remained within normal ranges and were comparable among all groups (Figure 6B). These findings collectively support the favorable systemic safety profile of the adoptively transferred macrophages during the treatment period.

**FIGURE 6:**
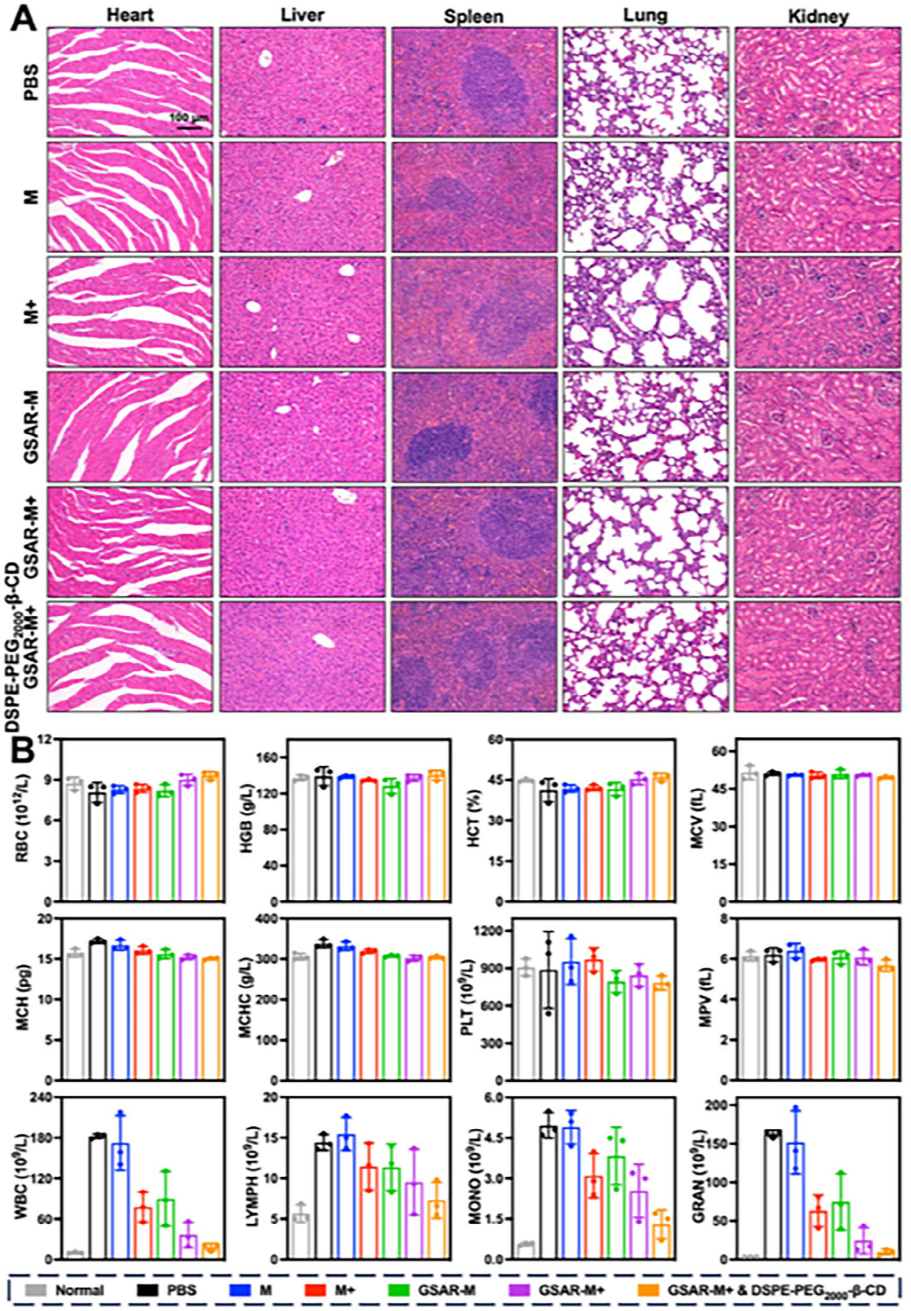
(A) Representative images of H&E‐stained organ tissue sections harvested from tumor‐bearing mice at the end of the treatment. (B) Hematological parameters measured at treatment termination, including RBC, HGB, HCT, MCV, MCH, MCHC, PLT, MPV, WBC, LYMPH, MONO, and GRAN (*n* = 3). All numerical data in these figures are shown as mean ± SD.

More importantly, we observed that the initially elevated systemic leukocyte populations in tumor‐bearing mice, including white blood cell (WBC), lymphocyte (LYMPH), monocyte (MONO), and granulocyte (GRAN) counts, exhibited a gradual normalization following treatment with the different macrophage formulations. Aligning with our efficacy evaluations, the most pronounced normalization of leukocyte profiles was achieved in the targeted group receiving the intratumoral pre‐injection of DSPE‐PEG_2000_‐β‐CD followed by the intravenous injection of GSAR‐M+, as evidenced by immune cell profiles that most closely resembled those of healthy controls. Ultimately, this normalization closely mirrors the observed reduction in tumor burden, confirming that the effective tumor clearance by the engineered macrophages subsequently resolves tumor‐induced hematological abnormalities.

## 3 Conclusion

In this study, we established a streamlined, cost‐effective, and safe non‐viral cell engineering approach by utilizing glycometabolic labeling to present adamantane moieties on the macrophage surface. This strategy successfully enables precise tumor targeting through bioorthogonal host‐guest recognition with β‐CD‐tagged cancer cells. Intriguingly, this glycometabolic labeling significantly upregulated activation markers, increased F‐actin content, and enhanced pseudopodia formation upon LPS stimulation, collectively leading to enhanced migration and superior phagocytosis of co‐cultured 4T1 cells, particularly those surface‐modified with β‐CD. The in vivo model further demonstrated that adoptively transferred macrophages subjected to the combined strategy of glycometabolic engineering and host‐guest recognition exhibited significantly improved tumor targeting efficiency, tumor suppression efficacy, and local immune microenvironment remodeling.

In contrast to conventional cell engineering approaches based on chimeric antigen receptor gene integration, the glycometabolic labeling approach harnesses intrinsic biosynthetic pathways to modify chemical tags on cell surfaces with superior efficiency, simplicity, and safety. While current in vivo applications of metabolic glycan labeling predominantly rely on azide‐modified sugars coupled with subsequent reagent‐free click chemistry, our validation of Ac_4_ManNAda‐based glycometabolic labeling on macrophages, integrated with bioorthogonal host‐guest recognition, substantially broadens the scope of glycometabolic labeling for adoptive cell therapy. Compared to covalent click chemistry, these non‐covalent supramolecular strategies exhibit superior association kinetics and harness multivalent interactions to achieve robust targeting avidity [58]. Combined with their inherent dynamic reversibility [59, 60], environmental responsiveness [61, 62, 63], and hierarchical programmable assembly [64, 65, 66, 67], this supramolecular strategy provides critical insights for developing next‐generation engineered immune cells toward precision therapy through adoptive cell therapy.

## 4 Experimental Methods

### 4.1 Materials

DSPE‐PEG_2000_‐β‐CD and Cy5.5‐NHS were purchased from Xi'an Ruixi Biological Technology. ManNAc, amantadine, adamantanecarboxylic acid, lecithin, cholesterol, and FITC were obtained from Macklin. Hoechst, Annexin V‐FITC/PI Apoptosis Detection Kit, Calcein/PI Live/Dead Viability Assay Kit, CFDA‐SE, 2‐NBDG, Crystal Violet Staining Solution, and Actin‐Tracker Red‐594 were purchased from Beyotime. FITC‐conjugated anti‐CD86 antibodies, FITC‐conjugated anti‐CD206 antibodies, PE‐conjugated anti‐CD80 antibodies, APC‐conjugated anti‐CD11b antibodies, and APC‐conjugated anti‐CD11c antibodies were purchased from BioLegend.

### 4.2 Cell Culture

RAW 264.7 cells and 4T1 cells were obtained from American Type Culture Collection (ATCC; USA). RAW 264.7 cells were cultured in DMEM medium (Gibco, USA) containing 10% fetal bovine serum (FBS) (Gibco, Brazil) and 1% streptomycin/penicillin (Gibco, USA). 4T1 cells were maintained in RPMI 1640 medium (Gibco, USA) supplemented with 10% FBS and 1% streptomycin/penicillin. All cells were cultured at 37°C in a humidified atmosphere with 5% CO_2_.

### 4.3 Preparation of Liposomes

Liposomes were produced using the classic film hydration method. Briefly, lecithin, cholesterol, and DSPE‐PEG_2000_‐β‐CD were dissolved in 5 mL of dichloromethane at a mass ratio of 9:4:4, along with an appropriate amount of FITC powder. The dichloromethane solvent was then evaporated under reduced pressure using a rotary evaporator, leaving a transparent film on the wall of the flask. The film was subsequently hydrated with 5 mL of deionized water under gentle rotation. After complete hydration, the suspension was repeatedly extruded through a filter membrane with a pore size of 200 nm to form CD‐FITC liposomes with uniform size. Finally, the prepared liposomes were dialyzed with deionized water to remove unencapsulated FITC.

### 4.4 Ac_4_ManNAda Metabolic Labeling Evaluation

The following method was applied to all metabolic labeling evaluation experiments in this study, and the procedure for Figure 2B,C is described here in detail as an example. Macrophages were seeded at a density of 5 × 10^5^ cells per well either in confocal dishes for imaging or in 12‐well plates for analysis by flow cytometry. After 12 h of incubation, cells were treated with Ac_4_ManNAda at concentrations ranging from 0 to 600 µm for 48 h. Subsequently, cells were rinsed with PBS and stained with CD‐FITC lipo for 5 min. For confocal imaging, cells in confocal dishes were rinsed twice with PBS to reduce background fluorescence, and images were obtained by confocal laser scanning microscopy (CLSM, Zeiss LSM710). To quantify the glycometabolic labeling efficiency, cells in 12‐well plates were collected and resuspended in PBS for analysis by flow cytometry using a CytoFLEX Flow Cytometer (Beckman Coulter). Data were further analyzed by FlowJo V10 software.

### 4.5 Cell Apoptosis Assay

Macrophages were seeded at a density of 5 × 10^5^ cells per well in 12‐well plates and cultured for 12 h. Subsequently, cells were incubated with Ac_4_ManNAda at concentrations ranging from 0 to 600 µm. Following 48 h of incubation, cells were washed and stained with Annexin V‐FITC and PI for 15 min in the dark. Apoptotic cells were analyzed using a CytoFLEX Flow Cytometer, and data were processed with FlowJo V10 software.

### 4.6 Live/Dead Cell Imaging

Macrophages were seeded at a density of 1 × 10^6^ cells per well in 6‐well plates and cultured overnight. After that, cells were incubated with Ac_4_ManNAda at concentrations ranging from 0 to 600 µm. Following 48 h of incubation, cells were rinsed and stained with Calcein‐AM and PI for 15 min in the dark. Subsequently, cells were rinsed twice with PBS to reduce background fluorescence, and images were obtained with a Leica DMi8 microscope.

### 4.7 Evaluation of Single‐Cell Mitochondrial Activity

To accurately assess single‐cell metabolic activity and rule out the interference of cytostatic effects, an MTT assay was performed across a precise cell number gradient. Unengineered macrophages (M) and engineered macrophages (GSAR‐M) were harvested, accurately counted, and seeded into 24‐well plates at specific density gradients of 1 × 10^6^, 2 × 10^5^, and 4 × 10^4^ cells per well. Following a 4‐h incubation to ensure complete cell attachment, whole‐well images were acquired using an Incucyte S3 Live‐Cell Analysis System to visually verify the uniform cell density between groups. The culture medium was then removed. The cells were subsequently incubated with 1 mL of MTT working solution per well for 1 h. Afterward, the unreacted working solution was carefully discarded, and the intracellular formazan crystals were fully dissolved by adding 1 mL of dimethyl sulfoxide to each well. The absorbance of the resulting solution was finally measured at 490 nm using a microplate reader for quantitative statistical analysis.

### 4.8 In Vitro Phagocytosis Assay

The following method was applied to all co‐culture experiments in this study, and the procedure for Figure 2H is described here in detail as an example. Macrophages were seeded at a density of 5 × 10^5^ cells per well in 12‐well plates and cultured overnight. After that, macrophages were incubated with 400 µm Ac_4_ManNAda for 48 h and then stained with Cy5.5‐NHS for fluorescence imaging or Hoechst for flow cytometry. Besides, CFDA‐SE‐stained 4T1 cells were incubated separately in serum‐free RPMI 1640 medium containing either PBS or 50 µm DSPE‐PEG_2000_‐β‐CD for 2 h. For the CD‐blocked group, CD‐tagged 4T1 cells were incubated with excess amantadine to occupy the cyclodextrin cavities before further co‐culture. The processed macrophages and processed 4T1 cells were then collected and co‐seeded in 12‐well plates in serum‐free medium at a 1:1 ratio, with a final density of 5 × 10^5^ cells per well. After 4 h of co‐culture, cells were rinsed twice with PBS to reduce background fluorescence and imaged using a Leica DMi8 microscope. To quantify the phagocytic efficiency, cells were collected and resuspended in PBS for flow cytometry using a CytoFLEX Flow Cytometer, and data were further analyzed by FlowJo V10 software. The phagocytic rate was determined as the proportion of double‐positive cells among the total of single‐positive and double‐positive cells.

### 4.9 Activation Marker Expression Assay

The following method was applied to all marker expression assays in this study, with the experiment for Figure 3A described here in detail as a representative example. Macrophages were seeded in 12‐well plates at a density of 5 × 10^5^ cells per well and divided into two temporal treatment groups. The first group was incubated in complete DMEM medium containing either PBS or 400 µm Ac_4_ManNAda. After 48 h of incubation, the medium was replaced with fresh complete DMEM and cells were cultured for an additional 48 h. The second group was maintained in complete DMEM for the initial 48 h, then incubated in complete DMEM medium containing either PBS or 400 µm Ac_4_ManNAda at the density of 5 × 10^5^ cells per well for the final 48 h. This design ensured that both groups were harvested for analysis at the same total time point (96 h after seeding). All processed cells were then collected, fixed with 4% paraformaldehyde for 20 min, and permeabilized with 0.1% Triton X‐100 for 15 min, followed by staining with FITC‐conjugated anti‐CD86 antibodies or FITC‐conjugated anti‐CD206 antibodies for 45 min. Finally, cells were washed twice with PBS for flow cytometry using a CytoFLEX Flow Cytometer, and data were further analyzed by FlowJo V10 software.

### 4.10 Glucose Uptake Assay

Macrophages were seeded in 12‐well plates at a density of 5 × 10^5^ cells per well and divided into two temporal treatment groups. The first group was incubated in complete DMEM medium containing PBS, 2 mm ManNAc, 400 µm Ada‐NH_2_, 400 µm Ada‐COOH, or 400 µm Ac_4_ManNAda. After 48 h of incubation, the medium was replaced with fresh PBS containing 10% FBS, and cells were starved for an additional 2 h, followed by incubation in PBS containing 10% FBS and 100 µm 2‐NBDG for 1 h. The second group was maintained in complete DMEM for the initial 48 h, then starved in fresh PBS containing 10% FBS for an additional 2 h, followed by a 1‐h incubation in PBS containing 10% FBS, 100 µm 2‐NBDG and one of the following: PBS, 2 mm ManNAc, 400 µm Ada‐NH_2_, 400 µm Ada‐COOH, or 400 µm Ac_4_ManNAda. All processed cells were then collected, washed twice with PBS, and analyzed using a CytoFLEX Flow Cytometer. Data were further analyzed with FlowJo V10 software.

### 4.11 Nitric Oxide Production Assay

To evaluate the functional polarization profile, NO production was quantified using the Griess assay. Macrophages were seeded and pre‐incubated with PBS, 2 mm ManNAc, 400 µm Ada‐NH_2_, or 400 µm Ac_4_ManNAda for 48 h. The culture medium was then replaced with fresh medium containing either PBS or 100 ng/mL LPS for an additional 24 h. During this period, cell morphology and density were monitored using an Incucyte S3 Live‐Cell Analysis System. Following the 24‐h incubation, the cell culture supernatants were collected into individual Eppendorf tubes. Griess reagent was added to each tube according to standard manufacturer protocols. The tubes were then photographed collectively to visually compare the colorimetric changes across different groups. Finally, the reacted solutions were transferred to a microplate, and the absorbance was measured at 540 nm using a microplate reader for the quantitative determination of NO concentration.

### 4.12 Real‐Time Imaging of Macrophage Morphological Changes

Macrophages were incubated in complete DMEM medium containing PBS or 400 µm Ac_4_ManNAda. Following 48 h of incubation, cells were collected and seeded in 12‐well plates at the density of 5 × 10^5^ cells per well, and maintained in serum‐free DMEM medium containing PBS or 100 ng/mL LPS for the subsequent 24‐h period. Throughout this time, cell morphology was monitored in real‐time using an Incucyte S3 Live‐Cell Analysis System placed in the cell culture incubator, with images acquired every 6 h. To eliminate the influence of cell reproduction across different groups on the results, the percentage of pseudopodia^+^ cells was determined as the ratio of cells with morphological changes to the total number of cells in the acquired images.

### 4.13 Transwell Migration Assay

Macrophages were incubated in complete DMEM medium containing PBS or 400 µm Ac_4_ManNAda. Following a 48‐h incubation, cells were collected and seeded in 12‐well plates at a density of 5 × 10^5^ cells per well and maintained for 24 h in serum‐free DMEM medium containing PBS or 100 ng/mL LPS. Subsequently, processed macrophages were collected and seeded in the upper chambers at a density of 5 × 10^4^ cells per chamber in serum‐free DMEM medium, while the lower chambers were filled with 600 µL complete DMEM medium. After 24 h, cells in the upper chambers were fixed with 4% paraformaldehyde for 20 min, and the cells on the inner side of the upper chambers were gently wiped off with cotton swabs, while the cells on the outer side were stained with crystal violet for 15 min. The stained cells were then photographed using a Leica K3C microscope, and the number of migrated cells in five randomly selected fields was counted.

### 4.14 F‐Actin Content Assay

The following method was applied to all F‐actin content assays shown in this study, and the procedure for Figure 4I,J is described here in detail as an example. Macrophages were incubated in complete DMEM medium containing PBS or 400 µm Ac_4_ManNAda. Following a 48‐h incubation, cells were collected and seeded in 12‐well plates at a density of 5 × 10^5^ cells per well and maintained for 24 h in serum‐free DMEM medium containing PBS or 100 ng/mL LPS. For fluorescence imaging, cells in 12‐well plates were rinsed with PBS, fixed with 4% paraformaldehyde for 20 min, and permeabilized with 0.1% Triton X‐100 for 15 min. After rinsing with PBS, cells were stained with Actin‐Tracker Red‐594 for 30 min and Hoechst for 10 min, washed twice with PBS, and then photographed with a Leica DMi8 microscope. To quantify the content of F‐actin in cells, cells in 12‐well plates were collected, fixed with 4% paraformaldehyde for 20 min, and permeabilized with 0.1% Triton X‐100 for 15 min. Subsequently, cells were rinsed with PBS, stained with Actin‐Tracker Red‐594 for 30 min, and washed twice with PBS for flow cytometry using a CytoFLEX Flow Cytometer, and data were further analyzed by FlowJo V10 software.

### 4.15 Animal and Tumor Models

#### 4.15.1 Animal

Female BALB/c mice aged 8 weeks were acquired from the Faculty of Health Sciences, University of Macau. All procedures were ratified by the Animal Ethics Committee, University of Macau (UMARE‐036‐2023).

#### 4.15.2 Subcutaneous Tumor Model

10^6^ 4T1 cells were inoculated into the right back of mice through subcutaneous injection.

### 4.16 In Vivo Biodistribution Analysis

When 4T1 tumor volumes reached approximately 200 mm^3^, tumor‐bearing mice were randomly divided into 5 groups (*n* = 3), with the last group receiving an intratumoral injection of DSPE‐PEG_2000_‐β‐CD 12 h prior to the intravenous administration of the respective formulation. Afterward, mice were intravenously injected with the respective macrophage formulations (M, M+, GSAR‐M, or GSAR‐M+). All macrophages were pre‐stained with Cy5.5‐NHS, and the fluorescence intensity of the cells was adjusted to a consistent level across all groups prior to intravenous injection. The targeting efficiency of the administered macrophages was evaluated using the IVIS imaging system (Lumina XR III) at time points of 12, 24, 36, and 48 h. At 48 h, hearts, livers, spleens, lungs, kidneys, and tumors of mice were collected for ex vivo imaging using the IVIS imaging system.

### 4.17 In Vivo Antitumor Therapy

When the 4T1 tumor volume reached approximately 50 mm^3^, tumor‐bearing mice were randomly divided into 6 groups (*n* = 5), with the last group receiving an intratumoral pre‐injection of DSPE‐PEG_2000_‐β‐CD 12 h prior to each of the three intravenous doses. Afterward, mice were intravenously injected with PBS, M, M+, GSAR‐M, or GSAR‐M+ every 4 days for 3 total doses. Body weight and tumor size were monitored every two days after the first dose. Mice were sacrificed at the end of the experiment, and tumors excised from the mice were photographed and weighed. The calculation formula for tumor volume was: volume = length × width × width × 0.5. Blood was collected immediately upon sacrifice of mice for hematology analysis. The harvested hearts, livers, spleens, lungs, kidneys, and tumors were prepared into 10 µm thick sections and stained for histological and immunohistochemical analysis.

### 4.18 Statistical Analysis

Data are presented as mean ± SD, with sample sizes (n) specified in figure legends. No outliers were excluded. Flow cytometry data were pre‐processed using FlowJo. Analyses were performed using GraphPad Prism. Significance was assessed using unpaired two‐tailed Student's t‐test for two groups, or one‐way and two‐way ANOVA for multiple groups, followed by Tukey's or Dunnett's post‐hoc tests with an alpha level of 0.05. Assumptions of normality and equal variance were validated using Brown–Forsythe or Bartlett's test where P is greater than 0.05.

## Author Contributions

**Ziyi Wang**: data curation, methodology, investigation. **Huazhong Yu**: investigation, resources, writing – review and editing. **Jianwen Wei**: methodology, investigation. **Caixia Yin**: writing – review and editing, resources, investigation. **Zhiqing Yang**: conceptualization, data curation, writing – original draft, methodology, validation, investigation. **Qingwen Zhang**: investigation, writing – review and editing, resources, supervision. **Qian Cheng**: conceptualization, writing – review and editing, investigation, validation, data curation, methodology. **Qun Guan**: methodology, investigation. **Ruibing Wang**: conceptualization, resources, writing – review and editing, supervision, funding acquisition, investigation.

## Conflicts of Interest

The authors declare no conflicts of interest.

## Supporting information




**Supporting File**: advs76468‐sup‐0001‐SuppMat.pdf.

## Data Availability

The data that support the findings of this study are available in the supplementary material of this article.
