# Supplementary material for: Artificial Host‐Guest Recognition Directs Glycometabolically Engineered Macrophages to Tumors
Source: Adv Sci (Weinh). 2026 Jul 13:e76468. Online ahead of print. doi: 10.1002/advs.76468 (PMC13360132; doi:10.1002/advs.76468)
Supplement: Supplementary file 1 — Supporting File: advs76468‐sup‐0001‐SuppMat.pdf. [file ADVS-9999-e76468-s001.pdf]

Supporting Information

Artificial Host-Guest Recognition Directs Glycometabolically Engineered Macrophages to Tumors

Zhiqing Yang§, Qian Cheng§, Ziyi Wang, Jianwen Wei, Qun Guan, Huazhong Yu, Qingwen Zhang, Caixia Yin* and Ruibing Wang**

§ These authors contribute equally to this work.

Supplementary figures

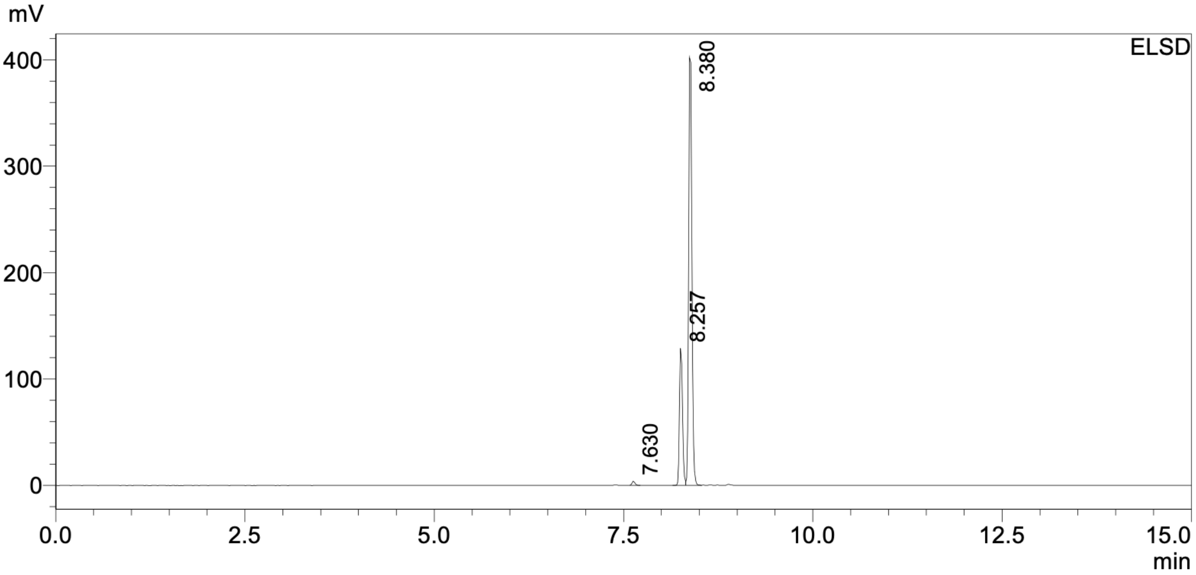

Figure S1: HPLC chromatograms of compound Ac_4ManNAda.

Ret. Time	Area	Area%
7.630	9773	0.631
8.257	354800	22.896
8.380	1185039	76.473

Table S1: Peak assignments in HPLC for Ac_4ManNAda isomers on the basis of retention times.

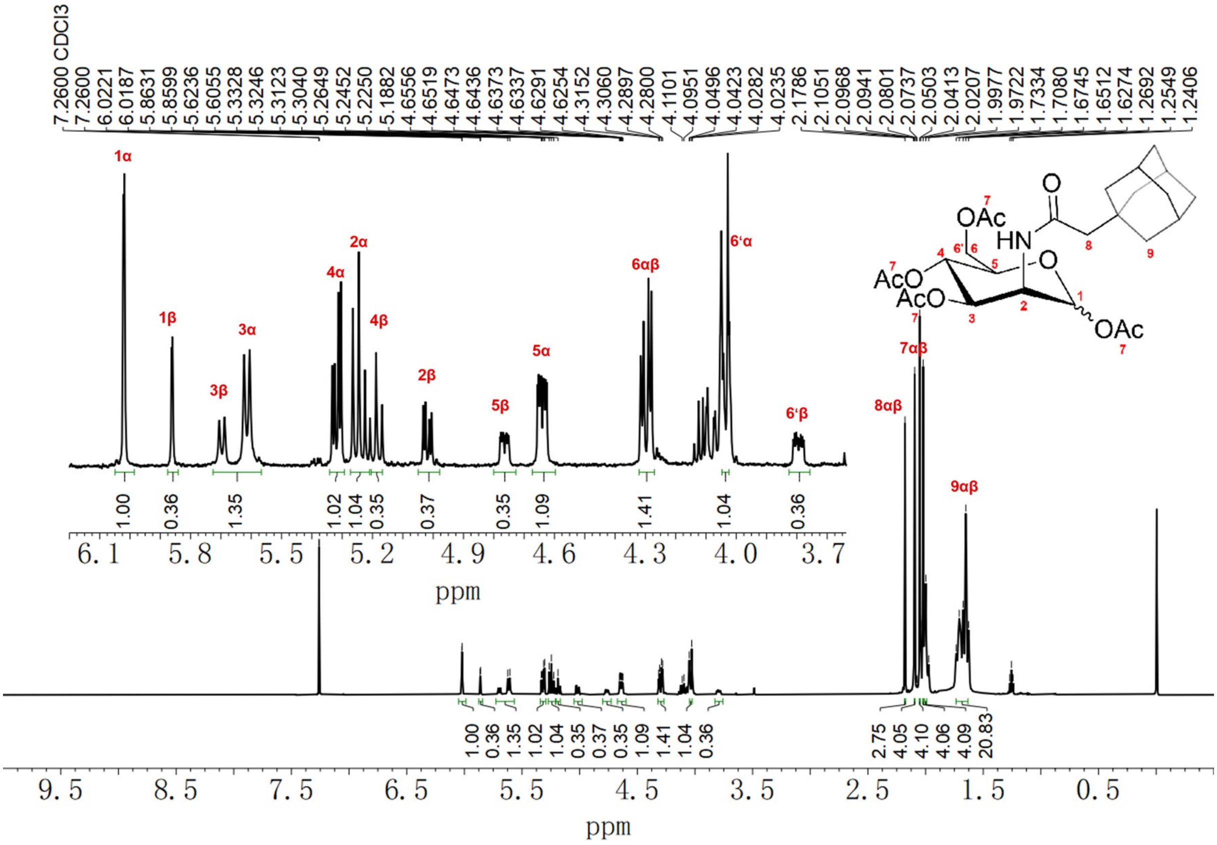

Figure S2: ^{1}H NMR spectrum (400 MHz, $CDCl_3$, 298 K) of compound Ac_4ManNAda.

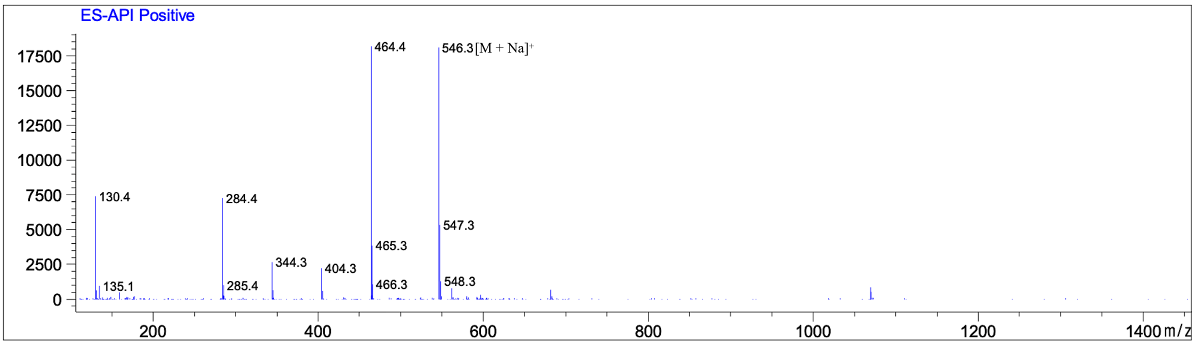

Figure S3: LC-MS spectrum of compound Ac_4ManNAda.

m/z	Assignment	Compound
546.3	$[M + Na]^+$	Ac_4ManNAda
464.4	$[M + H - HOAc]^+$	Ac_3ManNAda
404.3	$[M + H - 2HOAc]^+$	Ac_2ManNAda
344.3	$[M + H - 3HOAc]^+$	AcManNAda
284.4	$[M + H - 4HOAc]^+$	ManNAda

Table S2: Peak assignments in LC/MS on the basis of MS results from Ac_4ManNAda compound.

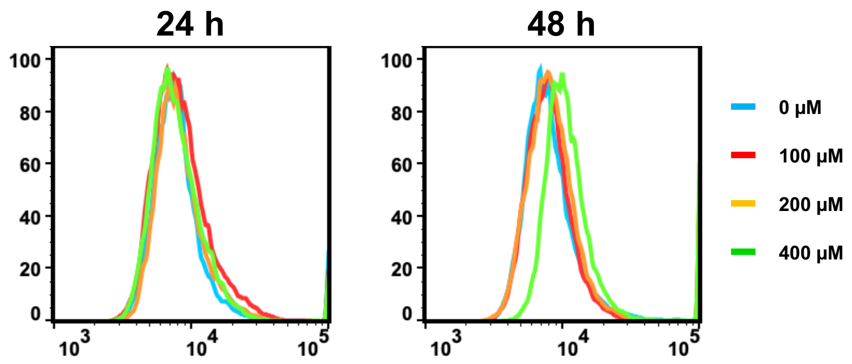

Figure S4: Flow cytometry analysis of macrophages following a 24-hour or 48-hour incubation with Ac4ManNAda at varying concentrations and subsequent 5-minute staining with CD-FITC lipo.

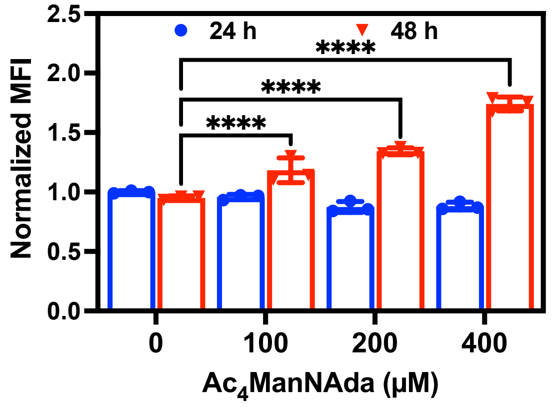

Figure S5: Normalized mean fluorescence intensities of macrophages following a 24-hour or 48-hour incubation with Ac4ManNAda at varying concentrations and subsequent 5-minute staining with CD-FITC lipo. Data are presented as mean ± SD (n = 3). Statistical significance was calculated using two-way ANOVA followed by Dunnett's multiple comparison tests. *P ≤ 0.05, P ≤ 0.01, ***P ≤ 0.001, ****P ≤ 0.0001.

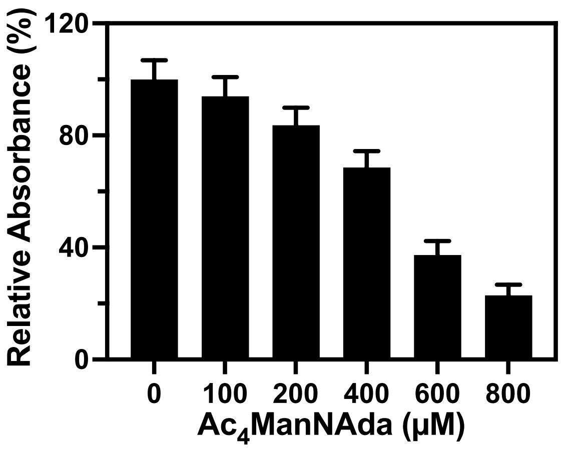

Figure S6: Cell viability assay. Macrophages were treated with indicated concentrations of $Ac_4ManNAda$ (0, 100, 200, 400, 600, and 800 μM) for 24 h. Cell viability is expressed as a percentage relative to the untreated control (0 μM).

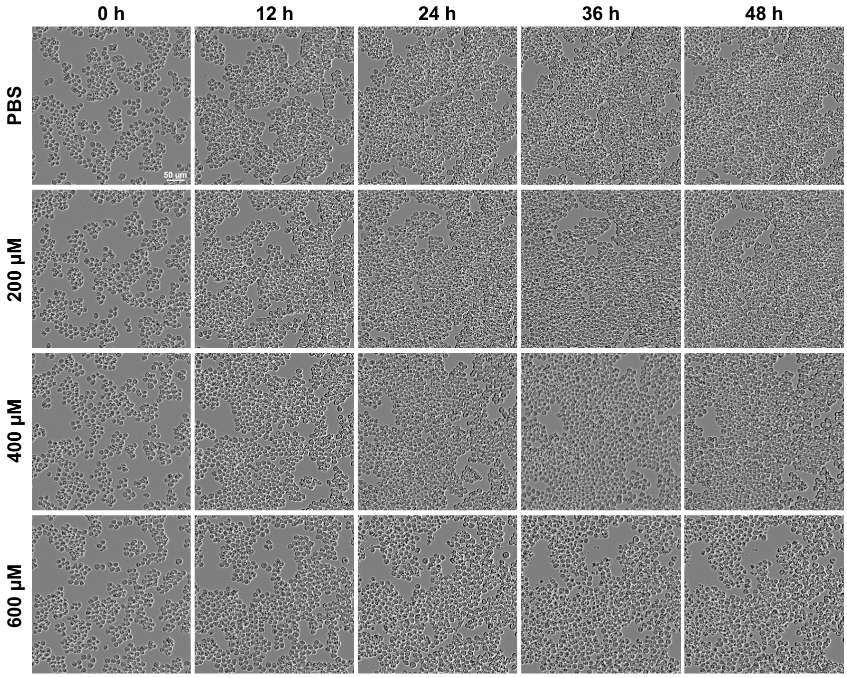

Figure S7: Real-time monitoring of macrophages over 48 hours upon incubation with 0, 200 μM, 400 μM, or 600 μM $Ac_4ManNAda$.

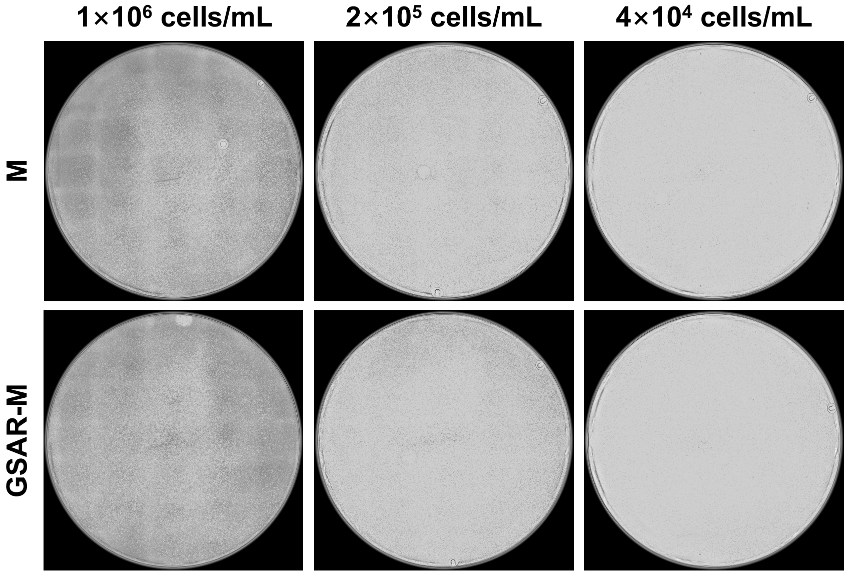

Figure S8: Representative whole-well images of M and GSAR-M captured by the Incucyte® S3 Live-Cell Analysis System following a 4-hour attachment period.

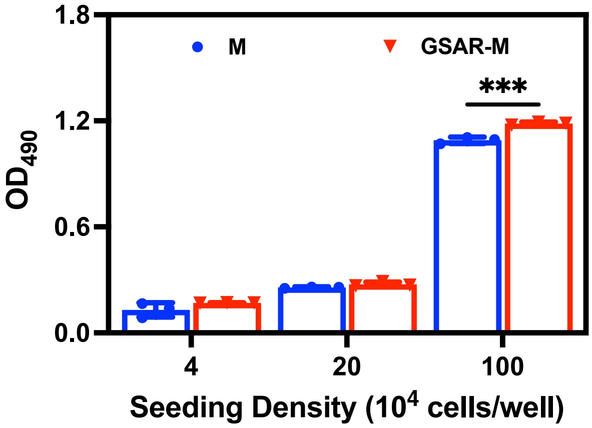

Figure S9: Quantitative evaluation of single-cell mitochondrial activity via an MTT assay normalized against a precise cell number gradient. The absorbance at 490 nm was measured for M and GSAR-M seeded at varied densities of 1×10^6, 2×10^5, and 4×10^4 cells per well to compare intrinsic metabolic activity at identical cell counts. Data are presented as mean ± SD (n = 3). Statistical significance was calculated using two-way ANOVA followed by Šídák's multiple comparisons test. * P ≤ 0.05, P ≤ 0.01, *** P ≤ 0.001, **** P ≤ 0.0001.

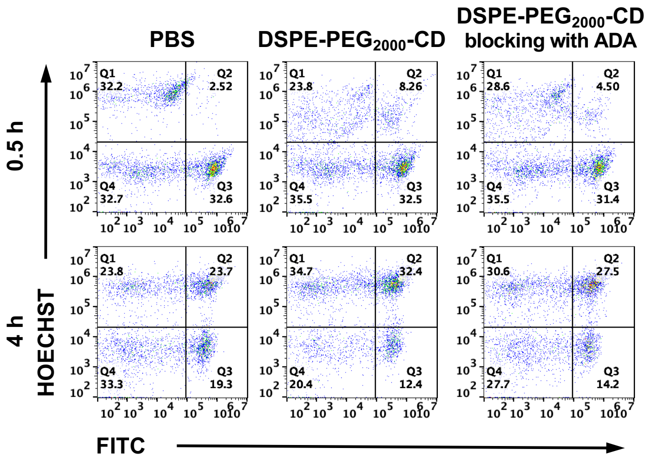

Figure S10: Representative flow cytometry scatter plots evaluating the targeting efficiency of GSAR-M co-cultured with unmodified, CD-tagged, or CD-blocked 4T1 cells for 4 hours.

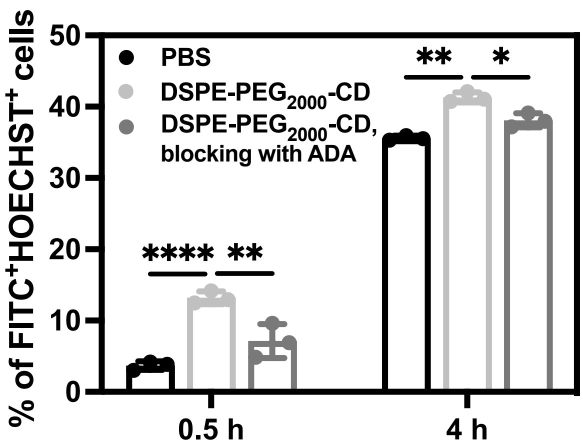

Figure S11: Quantitative analysis of the targeting efficiency represented by the percentage of double-positive events across different co-culture groups. Data are presented as mean ± SD (n = 3). Statistical significance was calculated using two-way ANOVA followed by Tukey's multiple comparisons test. * P ≤ 0.05, P ≤ 0.01, *** P ≤ 0.001, **** P ≤ 0.0001.

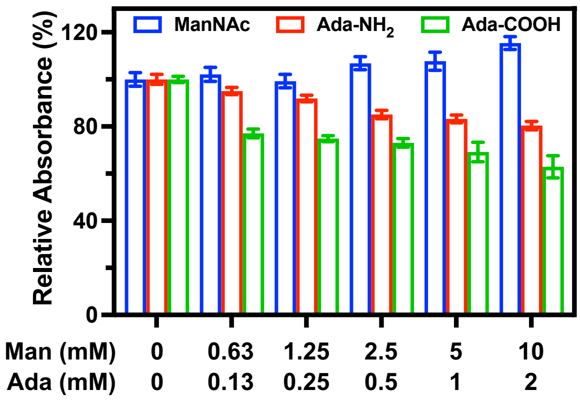

Figure S12: Quantitative evaluation of the relative absorbance of macrophages following 24-h incubation with extended concentration gradients of ManNAc, Ada-NH_2, and Ada-COOH using an MTT assay. The tested concentrations reached up to 10 mM for ManNAc and up to 2 mM for both Ada-NH_2 and Ada-COOH (n = 8).

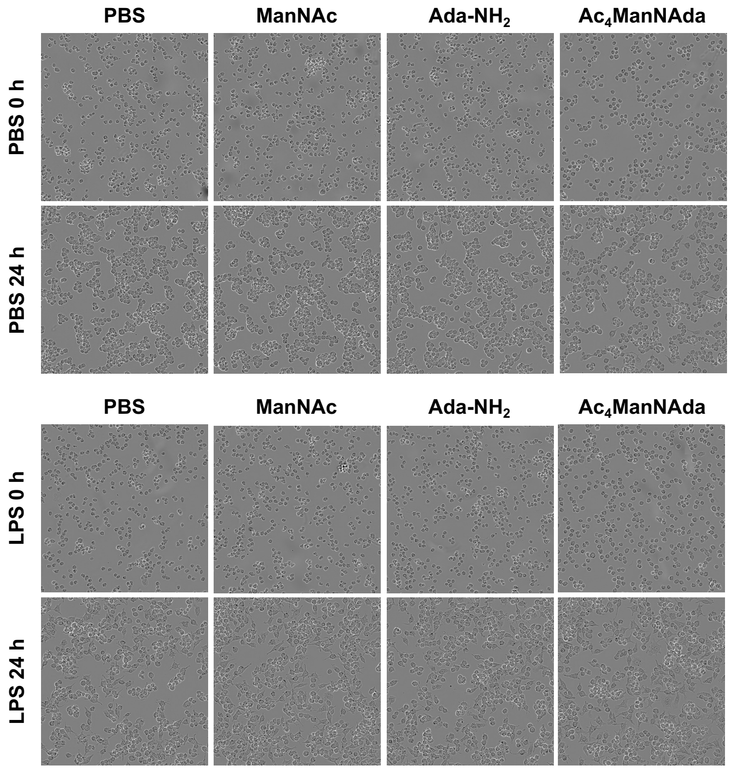

Figure S13: Representative images acquired by the Incucyte® S3 Live-Cell Analysis System showing the morphology and consistent cell density of macrophages pre-incubated with PBS, ManNAc, Ada-NH_2, or Ac_4ManNAda for 48 hours, followed by an additional 24-hour incubation with PBS or LPS.

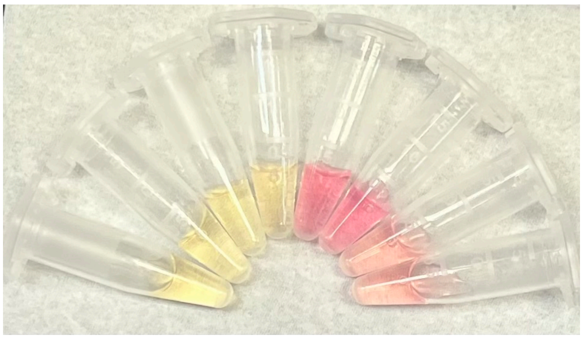

Figure S14: Macroscopic photograph demonstrating the visual colorimetric results of the Griess assay in Eppendorf tubes.

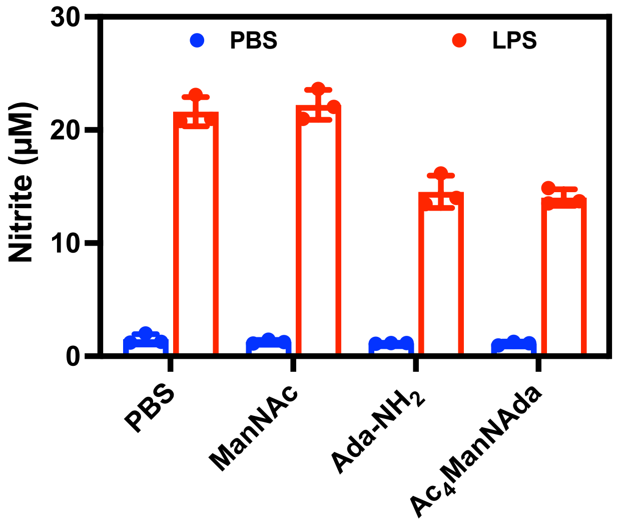

Figure S15: Quantitative evaluation of NO production in the cell culture supernatants measured by the Griess assay across different pre-incubation and subsequent stimulation conditions (n = 3).

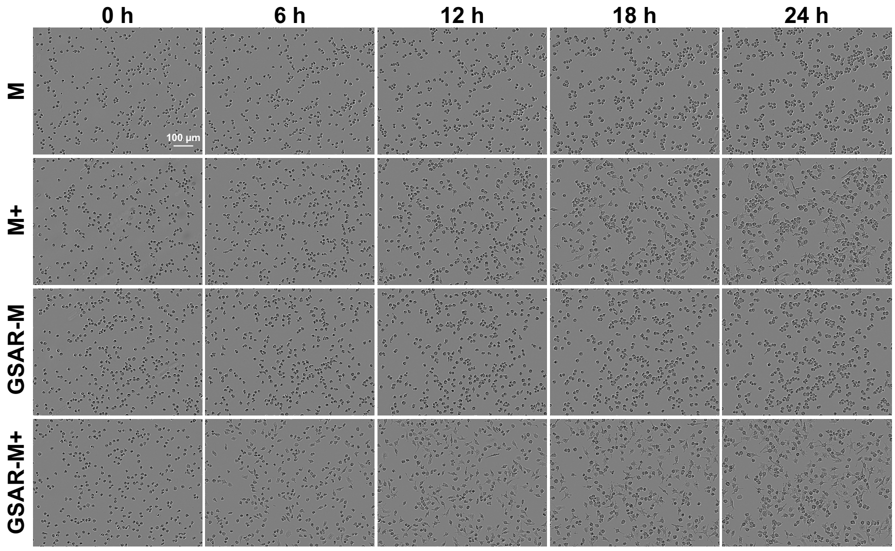

Figure S16: Real-time monitoring of M, M+, GSAR-M, and GSAR-M+ over 24 hours.
